# Integrated Physiological, Transcriptomic, and Metabolomic Analyses of the Response of Peach to Nitrogen Levels during Different Growth Stages

**DOI:** 10.3390/ijms231810876

**Published:** 2022-09-17

**Authors:** Yu Zhang, Jiying Guo, Fei Ren, Quan Jiang, Xin Zhou, Jianbo Zhao, Xin Liu

**Affiliations:** 1 Institute of Forestry and Pomology, Beijing Academy of Agriculture and Forestry Sciences, Beijing 100093, China; 2 Key Laboratory of Biology and Genetic Improvement of Horticultural Crops (North China), Ministry of Agriculture and Rural Affairs, Beijing 100093, China; 3 Beijing Engineering Research Center for Deciduous Fruit Trees, Beijing 100093, China; 4 Department of Pomology, College of Horticulture, China Agriculture University, Beijing 100193, China

**Keywords:** peach, nitrogen supply levels, growth period, transcriptome analysis, metabolome analysis

## Abstract

This study performed physiological, transcriptome, and metabolite analyses of peach fruit under different nitrogen (N) conditions at different growth stages. Nitrogen management directly affected the yield, fruit quality, and metabolites of peach in different growth stages. Different fertilizing time influenced yield and leaf N concentration. RNA-Seq was used to analyze the influence of N levels at the fruit pit hardening (PH) and fruit expansion (FE) stages. Kyoto Encyclopedia of Genes and Genomes (KEGG) pathway analysis revealed differentially expressed genes (DEGs) related to carbon and nitrogen metabolite processes. Metabolome analysis shows that applying different nitrogen fertilizers at different growth stages of peach mainly affected metabolites related to carbon and amino acids. This research provides insight into the metabolic processes underlying different N responses during different growth stages and provides a foundation to improve the efficiency of N use in peach.

## 1. Introduction

Nitrogen (N) is essential for plant growth as it is required for the synthesis of proteins, nucleic acids, phospholipids, chlorophyll, hormones, vitamins, and alkaloids [1]. Crop yield can be efficiently increased by effectively applying N fertilizer. However, excessive application of N fertilizer is a common phenomenon in China [2], especially during cultivation of vegetables and fruits with high economic benefits. N fertilizer input in typical plastic-shed vegetable fields was more than 1200 kg N ha^−1^ in Shouguang County, China, far greater than the actual crop demand [3]. In grapes, there has been poor development of rational fertilizer management strategies in vineyards with large amounts of N fertilizers (including inorganic and organic fertilizers) commonly applied to ensure high yields in North China [4], with nitrogen application rate (chemical fertilizer and animal manure) as high as 1140–5250 kg/hm^2^ [5]. The amount of N fertilizer applied in apple production has reached 600–800 kg/hm^2^, far exceeding the requirements of apple trees [6]. Excessive nitrogen application not only can cause serious environmental pollution, the excessive N will affect the carbon (C)−N balance and negatively impact soluble sugars, flavonoids, and other fruit quality indicators [7,8]. Therefore, proper nitrogen fertilizer management can optimize yield and quality.

Peach (*Prunus persica* (L) Batsch) is one of the most economically important fruit crops in the Rosaceae family, with broad climate range and relatively high yield [9]. Peach has served as a model species for genomic research of Rosaceae, making a comprehensive metabolomic study of this species imperative [10]. The production of new peach varieties to meet various demands of flavor or quality has been the goal of several peach breeding programs in various nations, which has led to considerable phenotypic variation. Various types of peach fruits exhibit different organoleptic and nutritional qualities, and these attributes are linked to the chemical make-up of the fruit. The fruit is one of the most metabolite-rich plant organs, with a wide variety of metabolites, including those involved in taste and flavor, those with nutritional or medicinal properties, and even those that allow plants to defend themselves against biotic and abiotic stress. There is significant variation in the sugar, acid, and amino acid content of various varieties of peach fruits [9,10,11].

The recent development and application of high-throughput sequencing, high-resolution mass spectrometry, and information processing technologies have facilitated systems biology (omics) strategies to explore major biological phenomena in plants. Transcript and metabolite datasets can be combined through correlation and clustering analyses and further represented as connection networks between genes and metabolites. For example, these approaches have been used to link gene expressions regulated by N, phosphorus (P), and potassium (K) deficiency to amino acid metabolism and energy production in tomato, as well as in lettuce leaves under N deficiency [12,13]. Transcriptomic and metabolomic analysis can be used to detect the status of different elements of plants and to guide appropriate fertilization programs.

There is significant genomic information and N metabolic data for rice and maize [14,15,16], but few studies in peach to examine changes in gene expression and metabolites under different N levels and fertilizing time. Therefore, there is a clear need to identify the genes and related metabolites that change under different N conditions to better understand N metabolism in peach during different growth stages.

In this study, we determined the physiological characteristics, as well as changes in transcription and metabolism under different N levels at different growth stages. The integrated analysis of peach transcriptome and metabolome provided insight into the carbon and nitrogen metabolism regulatory mechanisms in response to nitrogen availability. The goal of this work was to explore the relationship between rational N fertilizer management and the accumulation of primary and secondary metabolites to evaluate the impact of C–N balance on peach fruit quality. The results of this work provide new insights into understanding the mechanism and improving peach fruit quality under high N conditions.

## 2. Results

### 2.1. Different Effects of Nitrogen Fertilization on Plant Growth for Application during Different Growth Stages

The effects of nitrogen fertilization were tested for peach using application of four levels of N (N0, 0 kg N ha^−^^1^; N1, 100 kg N ha^−^^1^; N2, 200 kg N ha^−^^1^; and N4, 400 kg N ha^−^^1^) applied during the fruit pit hardening stage (PH) or the fruit expansion stage (FE). The results show that N levels and time of fertilization had highly significant effects on the yield of peach. However, the interaction between N level and fertilizing time had no significant effect on yield (*p* = 0.574; Table 1). When applied in the PH stage, nitrogen application in the range of 0–200 kg N ha^−^^1^ (N0–N2) increased yield with increasing nitrogen application, and the N2 treatment increased the yield by 53.08% compared with that under N0 treatment; however, under N3 treatment, the yield did not increase and did not significantly differ from the N0 treatment. Fertilization during the pit hardening stage increased yield by 14.96% compared with fertilization during the fruit expanding stage (Table 1).

A significant effect of fertilizing time was observed for leaf N concentration (*p* < 0.01; Table 1). Nitrogen application levels did not significantly affect leaf N concentration at two growth stages. The interaction between N level and fertilizing time had no significant effect on leaf N concentration (*p* = 0.876).

### 2.2. Nitrogen Affects Fruit Quality at Different Growth Stages

The total soluble solid content was significantly affected by N levels (*p* = 0.022) and the interaction between nitrogen level and fertilizing time (*p* = 0.041). Fertilizing time did not affect the total soluble solid content in fruit. With the increase in nitrogen application, the total soluble solid content of fruit increased. By the N3 treatment, compared with the N0 treatment, the solid value increased by 11.55%.(Table 1). VC content of fruit was unaffected by the timing of fertilization. However, the VC content of fruit was affected by the interaction between N level and the time of fertilization (Table 1).

### 2.3. Nitrogen Influences Differentially Expressed Genes at Different Growth Stages

Based on measurements of plant growth status and fruit quality, the period of N application in the orchard had a more significant effect on the growth of fruit trees than the levels of N application. Of the four nitrogen supply levels, N0 and N2 treatments had a greater effect, so these two nitrogen supply levels were selected for transcriptome and metabolome analysis.

**Figure 1:**
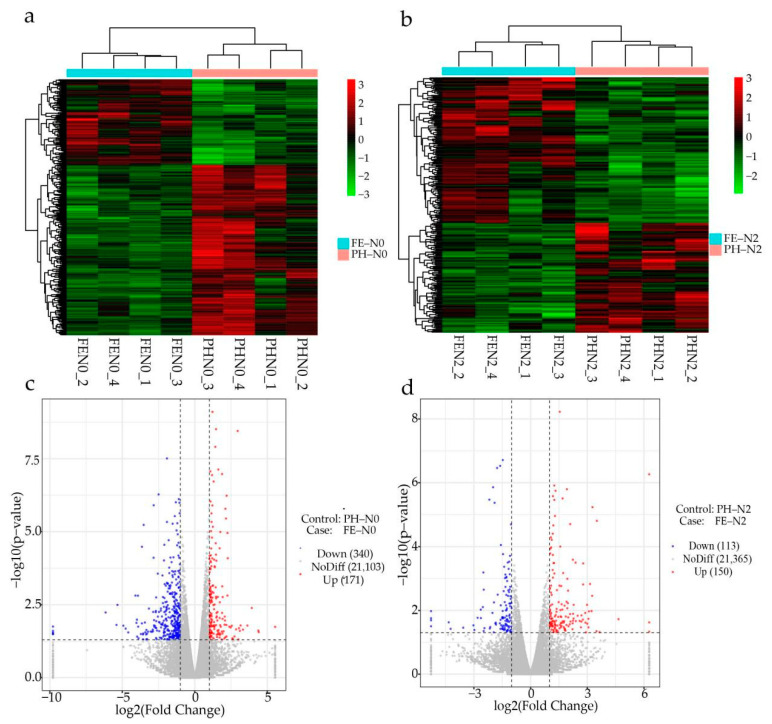
Different effects of nitrogen fertilization on transcriptome for application at different growth stages. (**a**,**b**) The cluster heat map and (**c**,**d**) volcano map of different genes in peach fruit under N0 and N2 at pit hardening stage (PH) and the fruit expansion stage (FE). PH-N0, 0 kg N ha^−^^1^ application at the fruit pit hardening stage; PH-N2, 200 kg N ha^−^^1^ application at the fruit pit hardening stage; FE-N0, 0 kg N ha^−^^1^ application at the fruit expansion stage; FE-N2, 200 kg N ha^−^^1^ application at the fruit expansion stage. The abscissa of heat map indicates the sample name and hierarchical clustering results, and the ordinate of heat map indicates differential genes and hierarchical clustering results. The blue dots of the volcano map represent down-regulated genes, the red dots represent up-regulated genes, and the gray dots represent undifferentiated genes; the numbers in parentheses represent the number of genes.

To identify DEGs under different N conditions at different growth stages in peach fruits, two transcriptomic comparisons were analyzed by RNA-Seq. For the DESeq2 analysis, |log2(Fold change)| ≥ 1 and a FDR (error detection rate) < 0.05 were used as screening conditions for paired comparisons to identify DEGs in the four treatment groups. These DEGs were then used to construct a cluster heat map. As shown, the time of fertilization treatment significantly affected the expression of different genes in the fruit at the same level of nitrogen (Figure 1a,b). The expression levels were investigated for a total of 21,614 genes, of which 511 were differentially expressed (171 genes up-regulated and 340 genes down-regulated) in fruit under N0 treatment (Figure 1c). A total of 21,628 transcripts of fruit were identified under N2 treatment, of which 263 genes were differentially expressed (150 genes up-regulated and 113 genes down-regulated) (Figure 1d).

**Figure 2:**
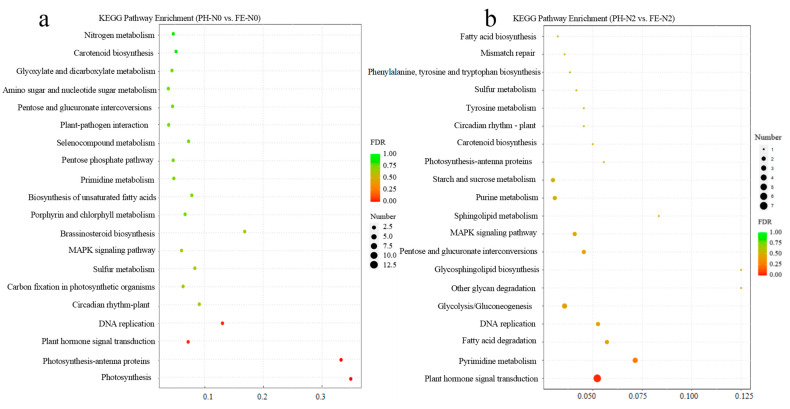
KEGG enrichment of different genes under different N conditions applied at different fertilizing times. (**a**) Statistics of KEGG enrichment under N0 in peach fruit. (**b**) Statistics of KEGG enrichment under N2 in peach fruit. PH-N0, 0 kg N ha^−1^ application at the fruit pit hardening stage; PH-N2, 200 kg N ha^−1^ application at the fruit pit hardening stage; FE-N0, 0 kg N ha^−1^ application at the fruit expansion stage; FE-N2, 200 kg N ha^−1^ application at the fruit expansion stage.

A KEGG pathway enrichment analysis was performed at PH-N0 vs. FE-N0 and PH-N2 vs. FE-N2 (Figure 2a,b). Enrichment analyses of the top 20 pathways showed that most identified DEGs were involved in nitrogen metabolism, photosynthesis, and carbon fixation in photosynthetic organisms at PH-N0 vs. FE-N0 in peach fruit (Figure 2a). The differential genes were annotated to the top 20 KEGG pathways, including starch and sucrose metabolism, plant hormone signal transduction, and primidine metabolism at PH-N2 vs. FE-N2 (Figure 2b).

### 2.4. Validation of DEGs by qRT-PCR

To validate the RNA-seq results, we used qRT-PCR to analyze the expression of twelve peach genes. The RT-qPCR analysis results are not significantly different from the RNA-Seq data, with similar trends observed in the up- and downregulated genes (Figure S1). These results confirm the reliability of the RNA-Seq results as indicative of actual transcriptome changes.

### 2.5. Metabolite Profiles of Fruit Response to Nitrogen Availability with Fertilization at Different Growth Stages

**Figure 3:**
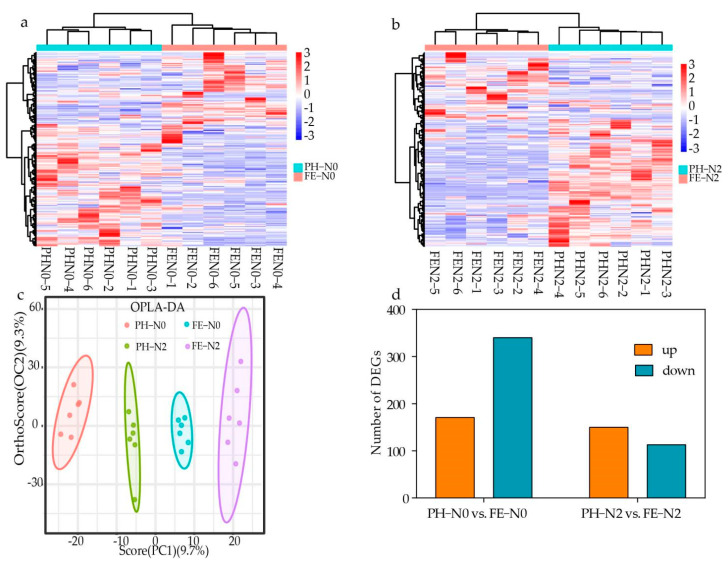
Different effects of nitrogen fertilization on metabolomic for application at different growth stages. (**a**,**b**) Heat map (**c**) OPLS-DA (**d**) Upregulation—downregulation metabolite bar chart of different metabolites in peach fruit fertilized during different growth stages using different nitrogen supply levels. PH-N0, 0 kg N ha^−^^1^ application at the fruit pit hardening stage; PH-N2, 200 kg N ha^−^^1^ application at the fruit pit hardening stage; FE-N0, 0 kg N ha^−^^1^ application at the fruit expansion stage; FE-N2, 200 kg N ha^−^^1^ application at the fruit expansion stage. The abscissa of heat map indicates the sample name and hierarchical clustering results, and the ordinate of heat map indicates differential metabolites and hierarchical clustering results.

To explore the effects of fertilizing time at different N supply levels, analyses were performed of differentially accumulated metabolites (DAMs) in PH-N0 vs. FE-N0 and PH-N2 vs. FE-N2. A heatmap of the identified DAMs was constructed, illustrating clear differences in the DAMs for these comparisons (Figure 3a,b). Orthogonal partial least-squares discriminant analysis (OPLS-DA) showed a 9.7% PC1 score and 9.3% Orthogonal T score (Figure 3c). These scores showed that different treatments corresponded to significant segregation in the OPLS-DA results. Analysis of the differential metabolites showed 108 DAMs for PH-N0 vs. FE-N0, with 34 metabolites up-regulated and 74 metabolites down-regulated, and 126 DAMs for PH-N2 vs. FE-N2, with 25 metabolites up-regulated and 101 metabolites down-regulated (Figure 3d).

**Figure 4:**
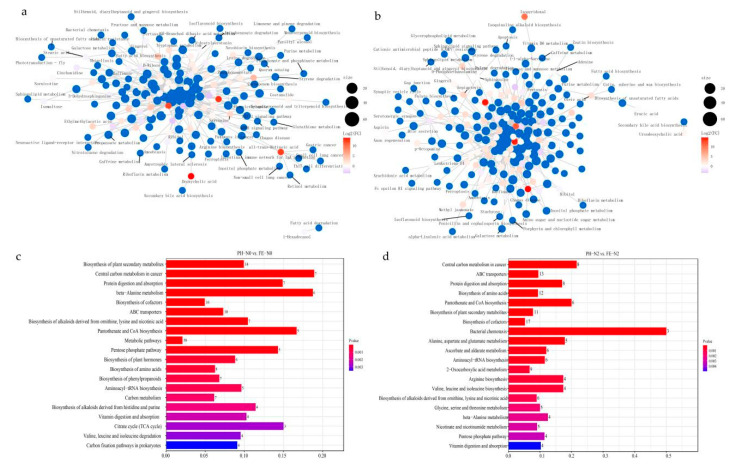
(**a**,**b**) KEGG enrichment network diagram (**c**,**d**) Top 20 KEGG pathways of differentially accumulated metabolites for peach fertilized in different growth stages using different nitrogen supply levels. PH-N0, 0 kg N ha^−^^1^ application at the fruit pit hardening stage; PH-N2, 200 kg N ha^−^^1^ application at the fruit pit hardening stage; FE-N0, 0 kg N ha^−^^1^ application at the fruit expansion stage; FE-N2, 200 kg N ha^−^^1^ application at the fruit expansion stage.

KEGG pathway enrichment was performed of the differential metabolites, and a network diagram was constructed (Figure 4a,b), in which the blue dots represent KEGG pathways and the other colored dots represent differential metabolites. The size of the blue dots represents the number of associated differential metabolites, and the colors of the other dots represent the size of their log2(FC) values. The enrichment analysis showed a total of 89 metabolic pathways in PH-N0 vs. FE-N0, with the top 20 impact values including biosynthesis of plant secondary metabolites, central carbon metabolism in cancer, protein digestion and absorption, beta—Alanine metabolism, biosynthesis of cofactors, and others (Figure 4c). Under PH-N2 vs. FE-N2 treatment, 111 KEGG pathways were enriched, and the top five were central carbon metabolism in cancer, ABC transporters, protein digestion and absorption, biosynthesis of amino acids, and pantothenate and CoA biosynthesis (Figure 4d).

### 2.6. Profiles of DEGs and DAMs in Carbon and Nitrogen Metabolism Pathways in Fruit under Different Fruit Development Stages

**Figure 5:**
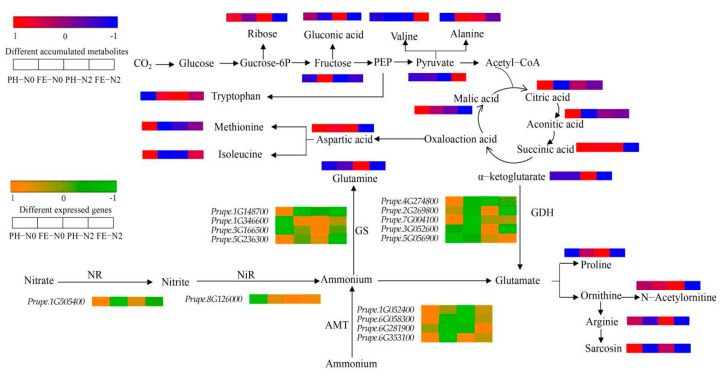
Differentially expressed genes and differentially accumulated metabolites associated with carbon and nitrogen metabolite pathways. NR, nitrate reductase; NiR, nitrite reductase; AMT, ammonium transporter; GS, glutamine synthetase; GDH, glutamate dehydrogenase. PH-N0, 0 kg N ha^−^^1^ application at the fruit pit hardening stage; PH-N2, 200 kg N ha^−^^1^ application at the fruit pit hardening stage; FE-N0, 0 kg N ha^−^^1^ application at the fruit expansion stage; FE-N2, 200 kg N ha^−^^1^ application at the fruit expansion stage.

Under different N conditions and different fruit development stages, amino acids and derivatives, carbohydrates, and organic acids exhibited great changes. Differences were seen in the levels of 20 different metabolites related to peach fruit carbon and nitrogen metabolism under different treatments (Figure 5). Concentrations of several key carbohydrates related to carbon (C) metabolism, including ribose and gluconic acid, were decreased in PH-N0 vs. FE-N0 and in PH-N2 vs. FE-N2, but fructose and pyruvate were increased. The levels of tricarboxylic acid (TCA) cycle intermediate citric acid, aconitic acid, succinic acid, α-ketoglutarate, and malic acid were decreased in PH-N0 vs. FE-N0 and PH-N2 vs. FE-N2. Among the 10 amino acids, alanine, tryptophan, proline, N-acetylornitine and glutamine were increased in PH-N0 vs. FE-N0 and valine, aspartic acid, isoleucine, sarcosine, and arginine were decreased. Similar changes were seen for aspartic acid, arginine, and sarcosine in PH-N2 vs. FE-N2 as in PH-N0 vs. FE-N0, but the other seven substances exhibited the opposite change.

We analyzed the changes in expression of genes associated with carbon and nitrogen metabolite pathways. The expression of nitrate reductase (NR) (*Prupe.1G505400*) and AMT genes (*Prupe.1G052400*, *Prupe.6G058300*, *Prupe.6G281900*, *Prupe.6G353100*) were down-regulated in PH-N0 vs. FE-N0. However, the expression of nitrite reductase (NiR) gene (*Prupe.8G126000*) was up-regulated in PH-N0 vs. FE-N0. Among the four glutamine synthetase (GS) genes, *Prupe.1G346600* and *Prupe.3G166500* gene expression was up-regulated and *Prupe.1G148700* and *Prupe.5G236300* expression was down-regulated in PH-N0 vs. FE-N0. Among the five GDH genes, only *Prupe.5G056900* expression was up-regulated, and the expression of *Prupe.4G274800*, *Prupe.2G269800*, *Prupe.7G004100*, and *Prupe.3G052600* was down-regulated. For PH-N2 vs. FE-N2, expression levels were down-regulated for NR (*Prupe.1G505400*), NiR (*Prupe.8G126000*), and GS (*Prupe.1G346600*, *Prupe.3G166500*, *Prupe.1G148700*, *Prupe.5G236300*). Among the four AMT genes, only the expression of *Prupe.6G353100* was down-regulated, with up-regulated expression of the other three genes (*Prupe.1G052400*, *Prupe.6G058300*, *Prupe.6G281900*) in PH-N2 vs. FE-N2. For the glutamate dehydrogenase (GDH) genes, the expression levels of *Prupe.4G274800*, *Prupe.7G004100,* and *Prupe.5G056900* were up-regulated and those of *Prupe.2G269800* and *Prupe.3G052600* were down-regulated in PH-N2 vs. FE-N2.

## 3. Discussion

Nitrogen is an essential nutrient for plant growth and development. However, excessive application of nitrogen has caused serious impacts on agricultural production and environment. Excessive N fertilization of rice, wheat, and corn has become common in China since 1980 [17]. Excessive N input poses a serious environmental threat, resulting in soil acidification [18], increased N_2_O and NO emissions [19,20,21], and large amounts of N leaching, including mineral N and dissolved organic N (DON), threatening groundwater quality and the health of local populations [22,23,24]. Additionally, high nutrient availability can actually restrict root growth and consequently rice production [17,25,26,27]. With rapid economic development and increasing demand for fresh fruit products in China, there is an increasing need for expanded fruit production, but fruit with a longer growth period requires higher N levels, and therefore takes up more N. Excessive application of nitrogen (N) fertilizer is common in Chinese apple production [2]. Excessive nitrogen application also occurs in peach orchards in China, and determination of appropriate nitrogen application strategies could reduce problems caused by excessive nitrogen application. Our results show that fruit yield decreased significantly under N3 treatment (Table 1). Additionally, fruit quality was not improved under N3 treatment (Table 1). Therefore, we determined that N3 treatment is excessive nitrogen application. The yield of peach reached maximum without sacrificing fruit quality under N2 treatment, indicating that N2 treatment is the most suitable level of nitrogen application for the late-maturing peach variety ‘2004-2-73’. Remarkably, we found that nitrogen application period had a greater impact on fruit quality than nitrogen application level (Table 1). To identify the underlying mechanisms related to plant responses to the appropriate amount of N application and the reasonable period of fertilization, we analyzed the affected physiological life processes under N0 and N2 treatments by measuring transcriptomic and metabolomic changes. KEGG pathway analysis revealed that many of the identified DEGs participated in photosynthesis, nitrogen metabolism, carotenoid biosynthesis, and carbon fixation in fruit of peach trees under different N conditions applied in different growth stages (Figure 2), indicating a clear effect of nitrogen stress on nitrogen metabolism in plants. Consistently, most amino acids and derivatives, nucleotides and derivatives, and lipids decreased under low nitrogen stress in apple leaves [28].

Dose and application strategy can affect plant response to applied nitrogen [29,30]. Different periods of N application affected the growth of mango trees (with changes in stem biomass, leaf count, and branch length) [31]. The development process of peach progresses through four recognized distinct stages (S1–S4) [32]. The first stage (S1) is the first exponential growth phase, and is characterized by a rapid increase in cell division and elongation. During the second stage (S2), the endocarp hardens to form the stone (pit hardening), with hardly any increase in fruit size; this is referred to as the pit hardening stage (PH) [33]. The third stage (S3) is the second exponential growth phase, with a rapid increase in fruit size and rapid cell division; in this study, this is the fruit expansion stage (FE). In the final stage (S4), the fruit reaches its final full size and enters the fruit ripening or climacteric stage [32]. There was a significant effect of fertilizing time on yield, N concentration of leaf between PH and FE stages (Table 1). There was also a significant effect of the interaction between nitrogen level and fertilizing time on the total soluble solid and VC content between these two stages (Table 1). We concluded that the timing of fertilizer application had a higher impact on late-ripening peach than the levels of nitrogen by combining the impacts of yield and fruit quality. With the proper timing of N application, yield and quality can be balanced. To identify the underlying mechanisms of the response to N fertilization, our study analyzed difference metabolites and differential genes in fruits for plants treated in PH and FE stages with the same amount of nitrogen. Under PH-N0 vs. FE-N0 treatment, the transcriptome KEGG enrichment pathways mainly included nitrogen metabolism, carbon metabolism and amino acid metabolism pathways (Figure 2a). The metabolome results were also the same, and the metabolite KEGG enrichment pathways involves carbon metabolism, nitrogen metabolism and amino acid metabolism pathways (Figure 4c). Similar results were obtained under PH-N2 vs. FE-N2 (Figure 2b and Figure 4d). The primary metabolic pathways impacted in our study were those for carbon and nitrogen (Figure 5).

C metabolism includes the TCA cycle and sugar, glycolysis and polyol metabolism, organic acid metabolism, and fatty acid metabolism [34]. Regardless of the nitrogen supply level, the content of fructose was significantly lower in PH stage than FE stage when the fruits were ripe, indicating that fructose was converted to other sugars. This result is consistent with the expected high FK/HK (fructose- and hexose-kinase) enzyme activities during the early growth stage of the fruit [35]. The TCA cycle is the most important source of energy for cells. Malic acid is a C4-dicarboxylic acid and a key intermediate of the tricarboxylic acid (TCA) cycle [36,37]. It had showed that malate was the most abundant accounting for the 62% of total organic acids, and suggesting its largest contribution to the overall peaches acidity [38,39]. Malic acid functions as a strong antioxidant in cells and can react with free radicals to inhibit lipid peroxidation and protect the mitochondrial membrane and mitochondrial function [40]. Apples have a built-in defense system to combat nitrogen stress, which includes increased malic acid content in the roots and maintenance of strong antioxidant status [28]. In our study, regardless of the level of nitrogen supply, fertilization during the pit hardening stage increased malic acid content in the fruit (Figure 5), suggesting that like in apple, malic acid improves the resistance of peach trees to nitrogen stress. Galacturonic acid is the most important esterified building component of pectins, polysaccharide constituents of middle lamella, primary and secondary cell walls in plants [38]. So it was higher in the pit hardening stage than in the fruit expanding stage, regardless of the nitrogen supply level (Figure 5). Amino acids play a key role in plants’ resistance to abiotic and biotic stresses. For example, the main classes of metabolites associated with temperature were amino acids and nucleotide metabolites, which have been reported to be involved in plant resistance to low temperature [41]. L-valine has been reported to be associated with stress [42,43]. In our study, valine was decreased under PH-N0 vs. FE-N0, indicating that the adaptation period of pit hardening stage to low nitrogen stress is higher than that of the fruit expansion stage.

In higher plant, inorganic nitrogen is absorbed and transported by specific transfer proteins, such as ammonium transporters (AMTs) and nitrate transporters (NR) [44]. In the cytosol, NO_3_^−^ is reduced to NO_2_^−^ by cytosolic NR and the produced NO_2_^−^ is further reduced to NH_4_^+^ by nitrite reductase (NiR) in plastids [45]. Glutamine synthetase (GS) uses NH_4_^+^ to make glutamine in the plastids [46]. At PH-N0 vs. FE-N0, expression of most NR, AMT and GDH genes decreased, which may lead to a decrease in the NR, AMT, and GDH enzyme activities, allowing accumulation of amino acid (such as glutamine). Glutamine accumulation was previously shown to inhibit the expression of nitrate reduction and transport genes [47].

## 4. Materials and Methods

### 4.1. Plant Materials and Growth Conditions

The experiment was conducted in 2019 at a peach orchard of Institute of Forestry and Pomology, Beijing Academy of Agriculture and Forestry Sciences, Pinggu District, Beijing, China (117.03° E, 40.12° N). Late-maturing peach ‘2004-2-73’ (bred by Institute of Forestry and Pomology, Beijing Academy of Agriculture and Forestry Sciences) was selected as the experimental material. Trees were planted in April 2012 at a density of 555 plant/hm^2^ (6 m between rows × 3 m between plants). The soil type at the study site is a calcareous alluvial fluvo-aquic soil with a loamy and silt texture. The soil has the following characteristics: 0–20 cm, 7.7 pH in water, 20.1 g/kg of organic matter, 1.11 g/kg of total nitrogen (N), 41.8 mg/kg of available phosphorus (P), and 20.6 mg/kg of total potassium (K). Four N fertilizer levels of 0, 100, 200, and 400 kg/hm^2^ N were tested, and the specific fertilization scheme is shown in Table 2.

Urea was applied to the soil all at once during the fruit pit hardening stage (PH) or the fruit expansion stage (FE). Urea was applied in the form of furrows, with two 20 cm deep trenches dug on each side of the tree, at a distance of 2.5 m from the tree. After each application, drip irrigation was used for immediate watering.

### 4.2. Plant Harvest and Determination of Fruit Quality Index

After ripening, the fruits were picked, weighed, and the yield was calculated. The leaf samples were heated at 105 °C for 30 min, dried to a constant weight at 75 °C, and ground into powder. Appropriate amounts of the ground plant materials were used to determine the total N concentration by a modified Kjeldahl digestion method.

The total soluble solids content was determined by handheld digital refractometer (PAL-1, Atago, Tokyo, Japan). Vitamin C (VC) content was measured according to GB 5009.86-2016 ‘Determination of ascorbic acid in Food.’

### 4.3. Transcriptome Analysis

We selected fruits that received N application in different periods for transcriptome analysis. Sample preparation for the transcriptome analysis and data analysis was performed at BioNovoGene (http://www.bionovogene.com/, accessed on 12 September 2022). Total RNA was isolated using the Trizol Reagent (Invitrogen Life Technologies, California, USA), and then the concentration, quality, and integrity were determined using a NanoDrop spectrophotometer (Thermo Scientific, Massachusetts, USA). Three micrograms of RNA were used as input material for the RNA sample preparations, and then sequencing libraries were generated according to the following steps. First, mRNA was purified from total RNA using poly-T oligo-attached magnetic beads. Fragmentation was carried out using divalent cations under elevated temperature in an Illumina proprietary fragmentation buffer. First strand cDNA was synthesized using random oligonucleotides and Super Script II, and second strand cDNA synthesis was subsequently performed using DNA Polymerase I and RNase H. The remaining overhangs were converted into blunt ends via exonuclease/polymerase activities and the enzymes were removed. After adenylation of the 3′ ends of the DNA fragments, Illumina PE adapter oligonucleotides were ligated to prepare for hybridization. To select cDNA fragments of the preferred 400–500 bp in length, the library fragments were purified using the AMPure XP system (Beckman Coulter, Brea, CA, USA). DNA fragments with ligated adaptor molecules on both ends were selectively enriched using Illumina PCR Primer Cocktail in a 15 cycle PCR reaction. Products were purified (AMPure XP system) and quantified by Agilent high sensitivity DNA assay on a Bioanalyzer 2100 system (Agilent, California, USA). The sequencing libraries were then sequenced on NovaSeq 6000 platform (Illumina, California, USA). Samples were sequenced on the platform to obtain9 image files, which are transformed by the software of the sequencing platform, and the original data in FASTQ format (Raw Data) are generated. Sequencing data contains a number of connectors, low-quality Reads, so we use Cutadapt software (v1.15, Rahmann S. et al., Dortmund, Germany) to filter the sequencing data to obtain high quality sequence (Clean Data) for further analysis. The reference genome and gene annotation files were downloaded from genome website. The filtered reads were mapping to the reference genome using HISAT2 (v2.0.5, Kim D. et al., Texas, USA). We used HTSeq (0.9.1, Planet E. et al., Barcelona, Spain) statistics to compare the Read Count values on each gene as the original expression of the gene, and then used FPKM to standardize the expression. Then, difference expression of genes was analyzed by DESeq (1.30.0, Anders S. et al., Heidelberg, Germany) with screened conditions as follows: expression difference multiple |log2FoldChange| > 1, significant *p <* 0.05. At the same time, we used R language Pheatmap (1.0.8, Ihaka R. et al., Auckland, New Zealand) software package to perform bi-directional clustering analysis of all different genes of samples. We obtained a heatmap according to the expression level of the same gene in different samples and the expression patterns of different genes in the same sample with Euclidean method to calculate the distance and Complete Linkage method to cluster. ClusterProfiler (3.4.4, Ihaka R. et al., Auckland, New Zealand) software was used to carry out the enrichment analysis of the KEGG pathway of differential genes, focusing on the significant enrichment pathway with *p <* 0.05. KEGG pathway enrichment analysis was performed using KOBAS software (http://kobas.cbi.pku.edu.cn/home.do, accessed on 12 September 2022) [48].

### 4.4. Metabolomics Analysis

Samples of peach treated with N0 and N2 (six replicates per treatment) were frozen with liquid nitrogen. Sample preparation for the metabonomic analysis and data analysis was performed at BioNovoGene (Suzhou, China) (http://www.bionovogene.com/, accessed on 12 September 2022). The analysis was carried out on a Waters ACQUITY UPLC instrument (Waters, Massachusetts, USA) equipped with an AB 4000 Triple Quadrupole Mass Spectrometer (AB 4000, Agilent, California, USA). The samples were separated through an ACQUITY UPLC^®^ BEH C18 column (2.1 × 100 mm, 1.7 μm; Waters, Massachusetts, USA), and the column temperature was 40 °C. The mobile phases were solvent A (1–0.1% formic acid) and solvent B (100% methanol). The flow rate was 0.25 mL/min, and the injection volume was 5 μL. The mass spectrometry conditions included an electrospray ionization (ESI) source and negative ion ionization mode. The ion source temperature was 500 °C, the ion source voltage was −4500 V, the collision gas was 6 psi, the curtain gas was 30 psi, and both the atomization gas and the auxiliary gas were applied at 50 psi. Multiple reaction monitoring (MRM) was used for scanning [49].

Through a principal component analysis, an orthogonal partial least squares discriminant analysis model and differentially accumulated metabolites (DAMs) were screened with FC (Fold change) ≥ 2 and VIP (variable importance in project) ≥ 1. Finally, the KEGG database was used for pathway enrichment analysis of DAMs.

### 4.5. qRT–PCR Analysis

A Revert Aid First Strand cDNA Synthesis Kit (Thermo Scientific, Waltham, MA, USA) was used to reverse transcribe 1 μg RNA with a CFX96 instrument (BioRad, Hercules, CA, USA) and SYBR^®^ Premix Ex Taq™ II (Takara, Dalian, China) to perform quantitative real-time reverse transcriptase PCR (qRT-PCR). The 2^−ΔΔCT^ method was used to compute the relative expression level of each gene. Four biological samples were used for all experiments. The primers used for qRT-PCR are listed in Table S1.

### 4.6. Statistical Analysis

Two-way ANOVA was conducted to test the effects of N levels, fertilizing time, and their interactions on yield, leaf N concentration, the total soluble solid, and VC content. Yield, N concentration, and fruit quality was determined for four replicates of each treatment. Metabolites were measured with six replicates for each treatment and transcriptome analysis was performed with four replicates for each treatment. The statistical analysis of the data from the control and different plant treatments was performed by analysis of variance (two-way ANOVA) using SPSS 20.0 software (Nie N.H. et al., California, USA. A probability value of *p* < 0.05 was considered a statistically significant difference. Data are presented as the mean standard deviation (SD) of different replicates.

## 5. Conclusions

The effects of different nitrogen supply levels on peach fruit physiology, transcriptome, and metabolites were analyzed for fertilization at different fruit development stages. Different N stresses influenced plant growth and physiological characteristics and treatment at different growth stages influenced DEGs and DAMs. Peach can respond to different environments through regulation of carbon and nitrogen metabolite pathways. The pit hardening stage of late-ripe peach is the most sensitive stage for nitrogen fertilizer treatment, likely because young fruit have a higher metabolite demand during cell division and rapid fruit growth. These results provide an improved understanding of the metabolic processes underlying different N responses in different fruit development stages. This provides a theoretical basis for nutrient management in peach orchard so that fruit farmers can use nitrogen fertilizer properly and avoid economic waste and environmental damage, balancing fertilization for soil and environmental health.

## Figures and Tables

**Table 1:** Different effects of nitrogen fertilization on plant growth and fruit quality for application at different growth stage.

		Yield (kg/plant)	N Concentration of Leaf (mg/kg)	The Total Soluble Solid (TSS) (%)	VC (Vitamin C) Content (mg/kg)
Fertilizing time (T)	PH	27.13 ± 1.41 A	2.82 ± 0.03 A	19.59 ± 0.42	0.45 ± 0.03
	FE	23.60 ± 1.67 B	2.67 ± 0.02 B	20.49 ± 0.39	0.38 ± 0.04
N levles (N)	N0	20.91 ± 2.38 b	2.76 ± 0.09	19.04 ± 1.12 b	0.43 ± 0.07
	N1	26.07 ± 3.85 ab	2.71 ± 0.06	19.74 ± 0.72 ab	0.40 ± 0.07
	N2	32.01 ± 1.87 a	2.78 ± 0.05	20.13± 0.63 ab	0.38 ± 0.08
	N3	22.34 ± 2.78 b	2.75 ± 0.11	21.24 ± 0.98 a	0.46 ± 0.11
Source of variation	T	0.028 *	0.003 **	0.069	0.063
	N	0.001 **	0.684	0.022 *	0.412
	T*N	0.574	0.876	0.041 *	0.001 **

Note: PH represents the application of nitrogen fertilizer during the fruit pit hardening stage; FE represents the application of nitrogen fertilizier during the fruit expanding stage. N0, 0 kg N ha^−1^; N1, 100 kg N ha^−1^; N2, 200 kg N ha^−1^; and N4, 400 kg N ha^−1^. The statistical results are of two-way ANOVA testing for the effects of N level and fertilizing time on the yield, leaf N concentration, the total soluble solid and VC content. * and **: statistically significant at *p* < 0.05 and *p* < 0.01, respectively. Uppercase letters indicate differences between fertilization times and lowercase letters indicate differences at different N levels.

**Table 2:** Fertilization scheme.

Treatment Name	N Supply (kg N ha^−1^)	Urea (g)	KH_2_PO_4_ (g)	KCl (g)
N0	0	0	77	124
N1	100	267	77	124
N2	200	534	77	124
N3	400	1070	77	124

## Data Availability

Not applicable.

## Supplementary Materials

The following supporting information can be downloaded at: https://www.mdpi.com/article/10.3390/ijms231810876/s1.
