# Supplementary material for: Integrated Physiological, Transcriptomic, and Metabolomic Analyses of the Response of Peach to Nitrogen Levels during Different Growth Stages
Source: Int J Mol Sci. 2022 Sep 17;23(18):10876. doi: 10.3390/ijms231810876 (PMC9505813; doi:10.3390/ijms231810876)
Supplement: Supplementary file 1 [file ijms-23-10876-s001.zip › ijms-1872781-supplementary.pdf]

Supplementary Materials

Table S1. Sequences of primers used in this study.

Gene name	Forward primer	Reverse prime
Prupe.6G141100	GATCAGTGCAGCAATGGAGGGTTAG	TCGCCACAGTCAACAAAGTCGTAG
Prupe.1G503100	TGCTGTGCTACAACTGTCTTCATCC	GTGCTTCACTCCCTCAACCTTTCC
Prupe.6G287600	ACACTGGAGACAACACCATCATTGC	CGTGAAGAATGACCACAACAGCAAC
Prupe.4G165800	CACCACAACACAAACAAACGCCTAG	CAAAGCAAACCAGCAGCAGCAG
Prupe.6G110800	CTACCCACACATTGCCTCACACAG	TCTGATGCCTGAGTAGCCTTGGAG
Prupe.2G211800	CTACCCACACATTGCCTCACACAG	TCTGATGCCTGAGTAGCCTTGGAG
Prupe.6G173300	CCTACGCCATTGGGAAACTCTAACC	GAGCCTCGGAGACCTGGACTTC
Prupe.6G173300	GTCTCTGCTTGGCTATCCAGTAACC	CACCTCATCAGACCAATCGCTAAGG
Prupr.3G032200	CCACCTCCTACACCTACTCCTTCTC	CAAGCCTGCCGTTGATCCTCTC
Prupe.3G032200	ACCGTCCTCCACACTTACTCTACTG	GCGGACTTGCTCGGGGATAATG
Prupe.1G276700	TTGACCTCTCTCAGCCTGTCTTCC	TGAGGACCAGGTTGAAGTAGGAGTG
Prupe.2G246800	GGCACCTCCTTCTGGAACTTCTTC	AGCACGGCAGGCGATATGATAAC
ACTIN	TGAGGTGAAAG	GGTGTGACGATGAAGAGTGATG

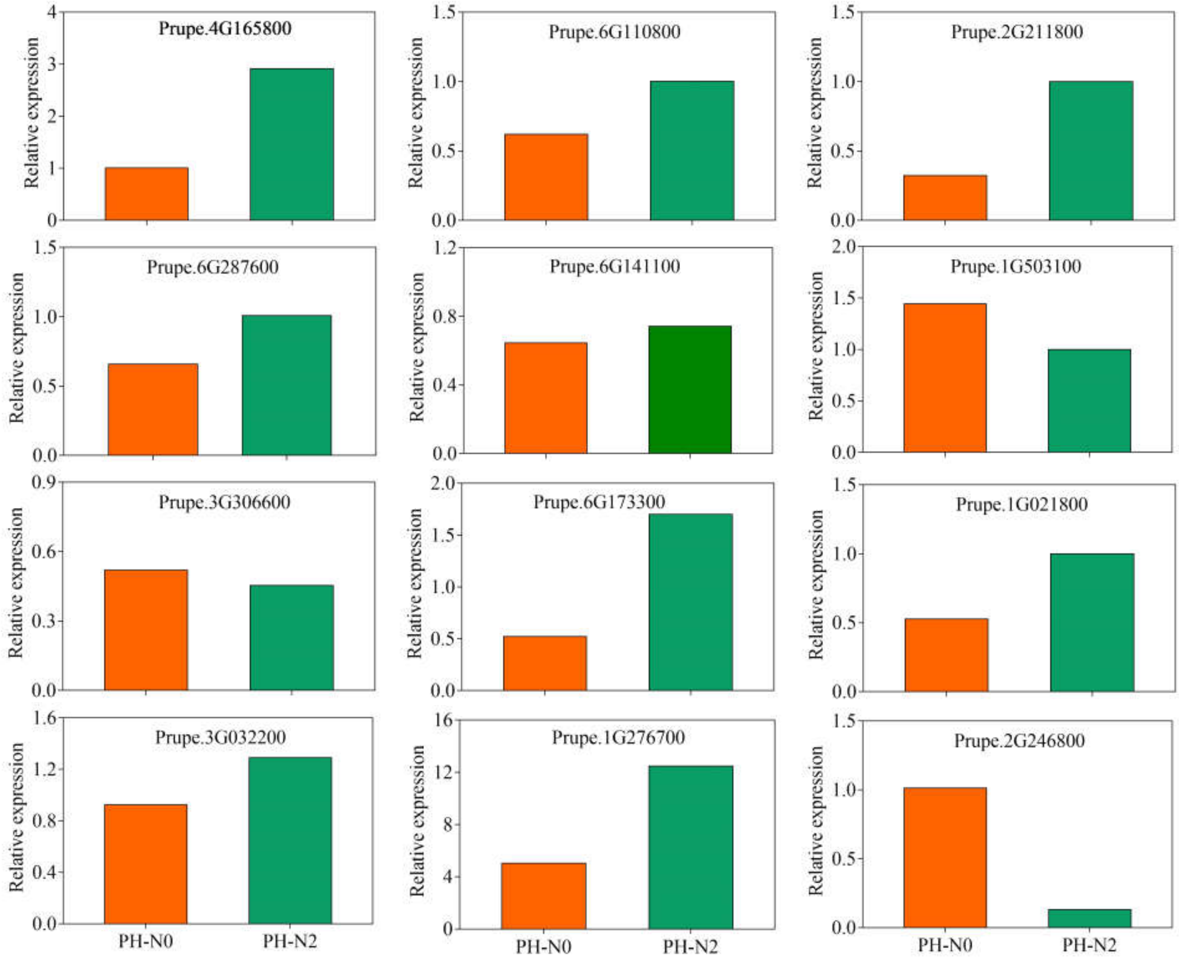

Figure S1. The relative expression of twelve peach genes.
